# Editorial: Computational Genomics and Structural Bioinformatics in Microbial Science

**DOI:** 10.3389/fgene.2022.850397

**Published:** 2022-02-11

**Authors:** Dhaval Acharya, Mohammed Kuddus, Saumya Patel

**Affiliations:** ^1^ Department of Microbiology, B. N. Patel Institute of Paramedical and Science, Gujarat, India; ^2^ College of Medicine, University of Hail, Hail, Saudi Arabia; ^3^ Department of Botany, Bioinformatics & Climate Change Impacts Management, School of Science, Gujarat University, Gujarat, India

**Keywords:** evolutionary and genomic microbiology, metagenomics, systems microbiology, microbiome data analytics, microbial bioinformatics

Microbes play a crucial roles in the lives of hosts (plants, animals, humans) and in almost any environment one can think of. The goal of this Research Topic was to gather a collection of high-quality original papers on the general theme of Computational Genomics and Structural Bioinformatics in Microbial Science. This Research Topic collection from *Frontiers in Genetics* brings together 11 articles focused on computational analysis of genomic microbiology as well as computational analysis of nucleotide or amino acid sequences and structures from genomic and metagenomic data.

The first paper by Bharathi *et al.* provides new insight into the understanding of *Methanobrevibacter ruminantium* M1 (MRU) growth physiology and lifestyle in the ruminants, and its potential to reduce anthropogenic greenhouse gas emissions worldwide. They have predicted and assigned a precise function to hypothetical proteins (HPs) and categorized them as metabolic enzymes, binding proteins, and transport proteins using a combined bioinformatics approach. Moreover, they propose new methane mitigation interventions that target the key metabolic proteins to reduce methane emissions in ruminants.

In the next paper, Choure *et al.*, elaborate on a comparative metagenomic analysis of two alkaline hot springs, Chhoti Anhoni and Badi Anhoni of Madhya Pradesh, India, and decoded the extremophiles for industrial enzymes. The objective of this study was to undertake, analyze, and characterize the microbiome to find out the inhabitant microbial population, and their functional characteristics. The study showed the presence of different unassigned bacterial taxa with great abundance, which indicates the potential of novel genera or phylotypes. Furthermore, the functional analysis of microbiomes revealed that most of the genes are associated with functions related to metabolism and environmental information processing.

Joshi *et al.* sequenced and analyzed the total number of 502 SARS-CoV-2 genomes from Gujarat, India to understand its phylogenetic distribution and variants against global and national sequences to understand its role in pathogenesis. The SARS-CoV-2 genomes they found, namely C28854T (Ser194Leu), showed an allele frequency of 47.62 and 7.25 percent in patients who dies from Gujarat and worldwide datasets, respectively, among the missense mutations. They concluded that SARS-CoV-2 genomes from Gujarat are forming distinct clusters under the GH clade of GISAID. Rampelli *et al.*, developed G2S, a bioinformatic tool for taxonomic prediction of the human fecal microbiome directly from the oral microbiome data of the same individual. This tool can be used in retrospective studies, where fecal sampling was not performed, especially in the field of paleomicrobiology.

Liu *et al.* developed a novel algorithm called DRAGoM for family-based ncRNA homology searches against metagenomic sequencing data (Detection of RNA using Assembly Graph from Metagenomic data). This tool can improve taxonomic analysis through facilitating the use of ncRNA families as taxonomic biomarkers. Andreu-Sánchez et al., benchmarked seven bioinformatic tools for genetic variant, calling in metagenomics data and evaluating their performance. This benchmark showed probabilistic tools that can be used to call metagenomes and recommendations of GATK’s tools as reliable variant callers for metagenomic samples.

Sevugapperumal et al. reported a draft genome sequence of B. amyloliquefaciens strain CB, which was isolated from the rhizospheric soil of a cotton plant, and which can be used as a reference sequence to explore and map specific genes related to antimicrobial peptide (AMP) genes and other important enzymes. The genome interpretation of B. amyloliquefaciens strain CB indicated antagonistic potential due to AMPs imparting various antifungal, antibacterial, and antiviral properties as well plant growth promotion, leading to strong prospects for uplifting sustainable agriculture.

Liu et al. attempted to reconstruct the biogeographical structure according to functional traits and the evolutionary lineage of B. amyloliquefaciens using comparative genomics analysis. Nimavat et al. analyzed 2,349 genome sequences of SARS-CoV-2 submitted in GISAID by a single institute pertaining to infections from the Gujarat state to know their variants and phylogenetic distributions with a major focus on the spike protein. The D614G variant in spike protein has been reported to have a very high frequency of >95% globally followed by the L452R and P681R.

Ahmad *et al.* modeled methyltransferase as antibiotics against foodborne pathogens. Its interactions were analyzed against a membrane protein of the Gram-positive and Gram-negative bacteria through *in silico* protein–protein interactions and established that it is a conclusively useful enzymobiotics agent.

The variety of the topic contributions by authors, including theoretical considerations and research articles, shed light on current advances in Computational Genomics and Structural Bioinformatics in Microbial Science and support further approaches for research in integrative microbial science.

